# *In Vivo* Protective Effects of Diosgenin against Doxorubicin-Induced Cardiotoxicity

**DOI:** 10.3390/nu7064938

**Published:** 2015-06-17

**Authors:** Chih-Tai Chen, Zhi-Hong Wang, Cheng-Chin Hsu, Hui-Hsuan Lin, Jing-Hsien Chen

**Affiliations:** 1 School of Nutrition, Chung Shan Medical University, Taichung City 40201, Taiwan; E-Mails: thomas.chen@unitybiotech.com (C.-T.C.); king@csmu.edu.tw (C.-C.H.); 2 Environment-Omics-Diseases Research Center, China Medical University Hospital, Taichung City 40402, Taiwan; E-Mail: rover_wang@hotmail.com; 3 School of Medical Laboratory and Biotechnology, Chung Shan Medical University, Taichung City 40201, Taiwan; 4 Clinical Laboratory, Chung Shan Medical University, Taichung City 40201, Taiwan

**Keywords:** doxorubicin, cadiotoxicity, diosgenin, antioxidant, cGMP

## Abstract

Doxorubicin (DOX) induces oxidative stress leading to cardiotoxicity. Diosgenin, a steroidal saponin of *Dioscorea opposita*, has been reported to have antioxidant activity. Our study was aimed to find out the protective effect of diosgenin against DOX-induced cardiotoxicity in mice. DOX treatment led to a significant decrease in the ratio of heart weight to body weight, and increases in the blood pressure and the serum levels of lactate dehydrogenase (LDH), creatine phosphokinase (CPK) and creatine kinase myocardial bound (CK-MB), markers of cardiotoxicity. In the heart tissue of the DOX-treated mice, DOX reduced activities of antioxidant enzymes, including superoxide dismutase (SOD) and glutathione peroxidase (GPx), were recovered by diosgenin. Diosgenin also decreased the serum levels of cardiotoxicity markers, cardiac levels of thiobarbituric acid relative substances (TBARS) and reactive oxygen species (ROS), caspase-3 activation, and mitochondrial dysfunction, as well as the expression of nuclear factor kappa B (NF-κB), an inflammatory factor. Moreover, diosgenin had the effects of increasing the cardiac levels of cGMP via modulation of phosphodiesterase-5 (PDE5) activity, and in improving myocardial fibrosis in the DOX-treated mice. Molecular data showed that the protective effects of diosgenin might be mediated via regulation of protein kinase A (PKA) and p38. Our data imply that diosgenin possesses antioxidant and anti-apoptotic activities, and cGMP modulation effect, which in turn protect the heart from the DOX-induced cardiotoxicity.

## 1. Introduction

Doxorubicin (DOX) belongs to the family of anthracyclines, and has been used to against cancer since late 1960s. It is the most effective anticancer drugs. However, studies of cardiotoxic effects of DOX have been reported [1,2]. Therefore, chemotherapy with DOX is limited by its cardiotoxicity. The development of cumulative dose-dependent cardiomyopathy may occur many years after the cessation of DOX treatment. It has been calculated that approximately 10% of patients treated with DOX will develop cardiomyopathy [3,4]. Multiple mechanisms are involved in DOX induced cardiomyopathy, such as the increase in cardiac oxidative stress and lipid peroxidation, and changes in adenylate cyclase activity leading to apoptosis and inflammation-related signaling pathway [5,6]. In previous research, free radical scavengers including probucol, vitamin E and ellagic acid have been demonstrated protection from DOX-induced cardiotoxicity, indicating the roles of reactive oxygen species (ROS) and nuclear factor kappa B (NF-κB) [7,8,9]. Acute DOX cardiotoxicity involves cardiomyocyte apoptosis. It is generally agreed that the elevated oxidative stress induced by DOX activates signaling pathway leading to cardiomyocyte apoptosis [10]. Caspase activity can be influenced by DOX, and caspase-3 activation is associated with DOX administration [11]. Thus, apoptosis plays a role in the development of heart failure via a loss of cardiomyocyte.

Due to the importance of DOX in the chemotherapy treatment for many types of cancer, strategies have been tried to prevent or attenuate the side effects of DOX administration, including the use of DOX analogues, alternative drug-delivery methods, and the iron-chelating agent. But so far, the ability of treatments to prevent or attenuate DOX-induced damage has been limited. Therefore, discovery of novel agents for reducing its side effects is still needed. Several studies in recent years suggest that sildenafil, a selective inhibitor of cyclic guanosine monophosphate (cGMP)-specific phosphodiesterase-5 (PDE5), may give some help against the DOX-induced cardiomyopathy [12,13]. PDE5 inhibitors have been shown to play a critical role against cardiac ischemia-reperfusion (I/R) injuries by activating cGMP-dependent protein kinase signaling pathway [14]. In the heart, cGMP modulates vascular tone, platelet function, cardiomyocyte contraction, mitochondrial function, and stress-response signaling [15].

Diosgenin is a steroidal saponin found in yam [16], the edible tubers of *Dioscorea opposita* and one of the most used plants in the world. Diosgenin has been shown to have favorable effects on anti-inflammatory [17], lipid metabolism [18], glucose lowering [19], and antioxidant activities [20,21]. Previous studies indicated that a supplementation of food rich in diosgenin, such as a yam variety (called air potato), has been shown to possess the protective effect on myocardial I/R injury in rats due to apoptosis and necrosis [22]. In the literature, diosgenin effectively protected against isoproterenol-induced myocardial necrosis in rats [23]. Moreover, the recent study found that diosgenin abrogated production of intracellular ROS [24]. Another finding suggests that diosgenin has a beneficial role against aortic remodeling induced by oxidative stress in diabetic state and decreases the lipid peroxidation in aorta [25]. Based on the potential role of diosgenin in ameliorating oxidative stress and injury, we attempted to evaluate the beneficial effects of diosgenin against the DOX-induced cardiotoxicity in mice.

## 2. Experimental Section

### 2.1. Chemicals

Doxorubicin hydrochloride and diosgenin were purchased from Sigma-Aldrich Co. (St. Louis, MO, USA). All chemicals used in this study were purchased from the commercially available.

### 2.2. Animals and Experimental Protocol

Male Balb/c mice, 4–5 weeks old, were obtained from National Laboratory Animal Center (National Science Council, Taipei City, Taiwan). Use of the mice was approved by the guidelines of the Instituted Animal Care and Use Committee of Chung Shan Medical University (IACUC, CSMU). Mice were housed on a 12-h light/dark cycle and fed with mouse standard chow diet (MF-18, Oriental Yeast Co., Ltd. Tokyo, Japan), and then started the experiments after 1-week acclimation. The mice were randomly divided into three groups (ten mice per group) and treated as follows: Group 1, vehicle (normal control); Group 2, DOX at 3 mg/kg of body weight once a week, i.p.; Group 3, DOX with diosgenin at 130 mg/kg of body weight once daily, p.o. (DOX + diosgenin). DOX was administered intraperitoneally to the mice of Groups 2 and 3 at a dose of 3 mg/kg once a week for 4 weeks (a total of 12 mg/kg). At the same time, Group 3 was treated with oral feeding of diosgenin at doses of 130 mg/kg daily for 4 weeks. The doses and injection regiments for these drugs were based on the reports published previously [26] with some modification. At the end of 4 weeks, mice were euthanized by carbon-dioxide asphyxiation followed by exsanguination. The hearts were excised and weighed, and serum and cardiac samples were collected and used for analysis as described below. 

### 2.3. Heart Rate, Blood Pressure Monitoring, and Blood Analysis

Heart rate and blood pressure was performed by tail cuff method using Blood Pressure Monitor for rats and mice (Model MK 2000- Muromachi Kikai Co. Ltd., Tokyo, Japan). Serum lactate dehydrogenase (LDH) activity was assayed by commercial kits (Randox, Crumlin, UK). Serum levels of creatine phosphokinase (CPK) and creatine kinase myocardial bound (CK-MB) were determined according to standard methods using diagnostic kits from BioSystems S.A. (Barcelona, Spain).

### 2.4. Measurement of Thiobarbituric Acid Relative Substances (TBARS), ROS and Antioxidant Status in Heart

TBARS (nmol/mg protein) level in cardiac tissue was determined by fluorescence spectrophotometer (excitation at 532 nm and emission at 600 nm) as described previously [27]. Quantification of TBARS was performed by comparison with a standard dosage of malondialdehyde (MDA), the lipid peroxidation product, which is generated by acid-catalyzed hydrolysis of 1,1,3,3-tetramethoxypropane. ROS in cardiac tissue was measured by using commercial kits (Calbiochem Inc., San Diego, CA, USA). Cardiac activities of glutathione peroxidase (GPx) and superoxide dismutase (SOD) were determined by commercial assay kits (Calbiochem Inc., San Diego, CA, USA), and glutathione (GSH) by commercial assay kits (OxisResearch, Portland, OR, USA).

### 2.5. Enzyme Immunoassay

Levels of cAMP and cGMP in the heart tissue were assayed with a competitive enzyme immunoassay (Cayman Chemical, Ann Arbor, MI, USA) according to the manufacturer’s protocol.

### 2.6. Real-Time Polymerase Chain Reaction (Real-Time PCR) for mRNA Expression

Total RNA was isolated from cells with a guanidinium chloride procedure as described previously, and the mRNA levels were analyzed by real-time quantitative RT-PCR using a Bio-Rad iCycler system (Bio-Rad, Hercules, CA, USA) [28]. The mRNAs were reverse-transcribed into cDNAs by using an iScript cDNA synthesis kit (Bio-Rad). The specificity of primers was tested by running a regular PCR for 40 cycles at 95 °C for 20 s and 60 °C for 1 min followed by electrophoresis on an agarose gel. The real-time PCR was performed using a SYBR supermix kit (Bio-Rad) and run for 40 cycles at 95 °C for 20 s and 60 °C for 1 min. Each 20-µL PCR mixture contained cDNA template, SYBR supermix kit, and 0.5 µM of each gene-specific primer. Specific primers were designed using Beacon Designer 2.0 software (Table 1). The PCR efficiency was examined by serial dilution of the cDNA, and the PCR specificity was checked by melting curve data. Each cDNA sample was triplicated and the corresponding no-RT mRNA sample was included as a negative control. The GADPH primers were included in every plate to avoid sample variations. The mRNA level of each sample for each gene was normalized to that of the GADPH mRNA.

**Table 1:** Sequences of different primers used for real-time PCR reactions.

Gene	Primer	Sequence
PDE5A	Forward	5′-AAATGGTGGGACCTTCACTG-3′
	Reverse	5′-GTGGCCGCTATCTTCTTCAG-3′
PDE3A	Forward	5′-AATGTGGCCGTATTCTGAGC-3′
	Reverse	5′-GAATCGGCTGTGTTGTGAGA-3′
NF-κB	Forward	5′-CAGACCGCAGTATCCATAGC-3′
	Reverse	5′-CGTGAAAGGGGTTATTGTTGG-3′
TGF-β	Forward	5′-TGACGTCACTGGAGTTGTACGG-3′
	Reverse	5′-GGTTCATGTCATGGATGGTGC-3′
GADPH	Forward	5′-TGTGTCCGTCGTGGATCTGA-3′
	Reverse	5′-TTGCTGTTGAAGTCGCAGGAG-3′

### 2.7. Protein Preparation and Western Blot Analysis

Proteins from the heart tissues were extracted in RIPA buffer (1% Triton X-100, 150 mmol/L NaCl, 5 mmol/L EDTA, and 10 mmol/L Tris-HCl, pH 7.0) containing a protease inhibitor cocktail. Protein extracts were subjected to centrifugation at 10,000 g for 10 min. Total protein (10–50 μg per lane) was electrophoresed and separated on 8%–15% SDS-poly-acrylamide gels and transferred to nitrocellulose membranes. After blocking with 5% nonfat dry milk, the membranes were incubated with the indicated primary antibodies overnight at 4 °C. The blots were incubated with the antibodies against caspase-3, phospho-PKA, PKA, phospho-p38, p38 and β-actin, purchased from Santa Cruz Biotechnology, Inc. (Santa Cruz, CA, USA). β-actin served as an internal control. The blot was quantified by enhanced chemiluminescence detection (Amersham Pharmacia Biotech, Little Chalfont, Bucks, UK).

### 2.8. Isolation of Mitochondria and Cytochrome C Assay

The preparation of cytosolic and mitochondrial fractions of cardiac tissue was performed as described previously [29]. The isolated hearts were washed in sterile PBS and the mitochondria were isolated according to the manufacturer’s instructions (Mitochondria Isolation Kit for Tissue; Pierce, Rockford, IL, USA). Briefly, tissues were minced after addition 800 mL Mitochondria Isolation Reagent A and carefully homogenized with 20 strokes on ice. The crude homogenates were then returned to the original tube, and 800 mL Mitochondria Isolation Reagent C was added. The tube was centrifuged at 700 g for 10 min at 48 °C. The supernatant was transferred and centrifuged at 3000 g for 15 min at 48 °C to obtain a more purified fraction of mitochondria. The resulting supernatant was transferred into a new tube and centrifuged at 12,000 g to produce a more purified cytosolic fraction and saved for cytochrome c assay. The pellet contained the isolated mitochondria. Mitochondria Isolation Reagent C (500 mL) was added to the pellet, and the mixture was centrifuged at 12,000 g for 5 min. The mitochondrial pellet was resuspended in 300 mL of mitochondria isolation buffer containing 0.1% Triton X-100 and protease inhibitors. Protein concentrations of both mitochondrial and cytosolic lysates were determined using BCA Protein Assay Reagents (Pierce, Rockford, IL, USA). To detect cytochrome c release into the cytosol, Western blotting was performed with antibodies against cytochrome c and COX IV, a mitochondrial marker (Santa Cruz, CA, USA).

### 2.9. Statistical Analysis

Results from ten mice (*n* = 10) were analyzed and expressed as means ± SD. Statistical analysis was done using one-way ANOVA, and *post hoc* comparisons were carried out using Duncan’s multiple-range test. *p* < 0.05 was considered statistically significant.

## 3. Results

### 3.1. Diosgenin Diminished DOX-Induced Losses of Heart Weight, the Ratio of Heart Weight to Body Weight, and Heart Rate, and Attenuated DOX-Increased Blood Pressure, and Serum Levels of Cardiotoxicity Markers

Effect of DOX on heart weight, body weight, and the ratio of heart weight to body weight are shown in Table 2. The heart weight and the ratio of heart weight to body weight were significantly decreased by DOX treatment, but diosgenin treatment diminished the DOX-induced loss in heart weight by 48.7% (*p* < 0.05). The decrease in the ratio of heart weight to body weight due to DOX treatment was also alleviated by diosgenin in the DOX plus diosgenin group comparing to the DOX group (*p* < 0.05). Heart rate and blood pressure were performed after the four weeks of DOX administration. Table 2 also shows that DOX treatment resulted in significant cardiac functional deterioration as characterized by increase in blood pressure and lower heart rate as compared with normal group (*p* < 0.05). LDH, CPK, and CK-MB are important clinical markers of cardiac injury [30]. As expected, serum levels of LDH, CPK and CK-MB were significantly elevated in the DOX alone treated group as compared with the control (*p* < 0.05). Treatment with diosgenin in the DOX plus diosgenin group significantly reduced their levels as compared with the DOX alone treated group (*p* < 0.05).

**Table 2:** Effect of diosgenin on body weight, heart weight, ratio of heart weight to body weight, heart rate, blood pressure, and serum levels of lactate dehydrogenase (LDH), creatine phosphokinase (CPK) and creatine kinase myocardial bound (CK-MB) in doxorubicin (DOX)-treated mice.

	Normal	DOX	DOX + Diosgenin
Body weight (g)	28.00 ± 0.00 ^b^	20.77 ± 3.64 ^a^	21.73 ± 2.84 ^a^
Heart weight (mg)	166.43 ± 15.82 ^b^	131.78 ± 21.74 ^a^	148.05 ± 15.51 ^ab^
Heart weight/body weight ratio	5.94 ± 0.57 ^b^	4.66 ± 1.05 ^a^	5.83 ± 0.75 ^b^
Heart rate (bpm) ^#^	611.40 ± 15.65 ^b^	566.31 ± 27.36 ^a^	595.59 ± 32.83 ^ab^
Blood pressure (mmHg)	98.08 ± 11.40 ^a^	119.93 ± 26.25 ^b^	98.88 ± 15.49 ^a^
Serum LDH (U/L)	173.02 ± 8.70 ^a^	263.91 ± 54.35 ^b^	152.13 ± 29.25 ^a^
Serum CPK (U/L)	106.31 ± 17.06 ^a^	241.22 ± 18.77 ^b^	165.32 ± 16.02 ^ab^
Serum CK-MB (U/L)	86.90 ± 6.72 ^a^	154.28 ± 15.03 ^b^	107.15 ± 10.33 ^ab^

Values are mean ± SD, *n* = 10. ^a,b^ Means in a row without a common letter differ, *p* < 0.05. ^#^: beats per minute.

### 3.2. Diosgenin Restored DOX-Induced Alterations in Oxidative Status

To confirm the induction of oxidative stress by DOX, tissue lipid peroxidation, antioxidation and antioxidant enzymes were also evaluated. As shown in Table 3, DOX treatment increased TBARS and ROS levels, decreased GSH content as well as GPx and SOD activities in the heart. As compared with DOX alone, diosgenin treatment lowered ROS and MDA levels, retained GSH content, and recovered cardiac GPx and SOD activities (*p* < 0.05) (Table 3).

**Table 3:** Effect of diosgenin on cardiac levels of thiobarbituric acid relative substances (TBARS) and reactive oxygen species (ROS), and glutathione (GSH), and activities of glutathione peroxidase (GPx) and superoxide dismutase (SOD) in DOX-treated mice.

	Normal	DOX	DOX + Diosgenin
TBARS (nmol/mg protein)	0.16 ± 0.02 ^a^	0.27 ± 0.07 ^b^	0.17 ± 0.04 ^a^
ROS (RFU/mg protein)	1.02 ± 0.16 ^a^	1.51 ± 0.50 ^b^	1.05 ± 0.16 ^a^
GSH (nmol/mg protein)	5.97 ± 0.28 ^b^	2.36 ± 0.99 ^a^	4.43 ± 1.37 ^ab^
GPx (U/mg protein)	43.79 ± 0.73 ^b^	27.52 ± 3.42 ^a^	40.19 ± 4.75 ^b^
SOD (U/mg protein)	4.29 ± 0.64 ^b^	2.90 ± 0.92 ^a^	3.94 ± 0.51 ^ab^

Values are mean ± SD, *n* = 10. ^a,b^ Means in a row without a common letter differ, *p* < 0.05.

### 3.3. Diosgenin Improved DOX-Regulated Levels of cAMP and cGMP, and mRNA Expression of PDE5A

**Figure 1:**
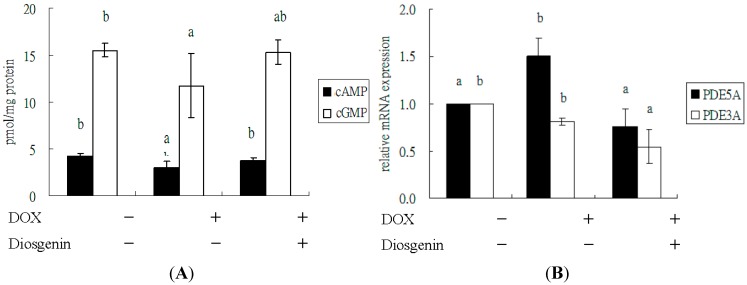
Effect of diosgenin on levels of cAMP and cGMP (**A**), and mRNA expressions of PDE5A and PDE3A (**B**) in heart tissues of mice treated with DOX for four weeks. Values are mean ± SD, *n* = 10. ^a,b^ Means in a row without a common letter differ, *p* < 0.05.

It is well known that cGMP exerts its effects by interacting with PDE, and cGMP-dependent signaling plays an important protective role in cardiac injury [14,15]. As compared with the control, treatment with DOX decreased the levels of cAMP and cGMP in the heart, especially the later (*p* < 0.05) (Figure 1A). Diosgenin augmented the cGMP and cAMP levels as compared with DOX alone (*p* < 0.05). Moreover, the cardiac mRNA expression of PDE5A was increased after a four-week treatment of DOX, whereas that of PDE3A was slightly affected. Treatment with diosgenin also attenuated both mRNA expressions compared to DOX alone (*p* < 0.05) (Figure 1B).

### 3.4. Diosgenin Weakened DOX-Induced Caspase-3 Activation and NF-κB Expression

**Figure 2:**
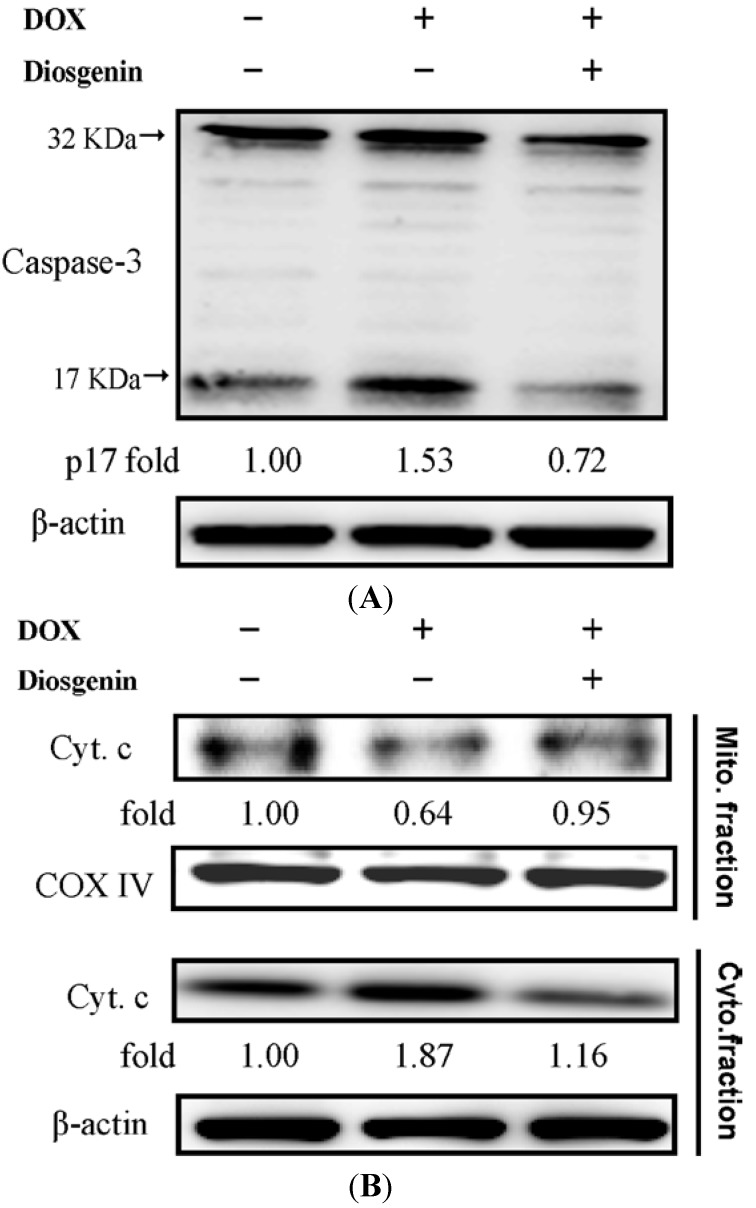

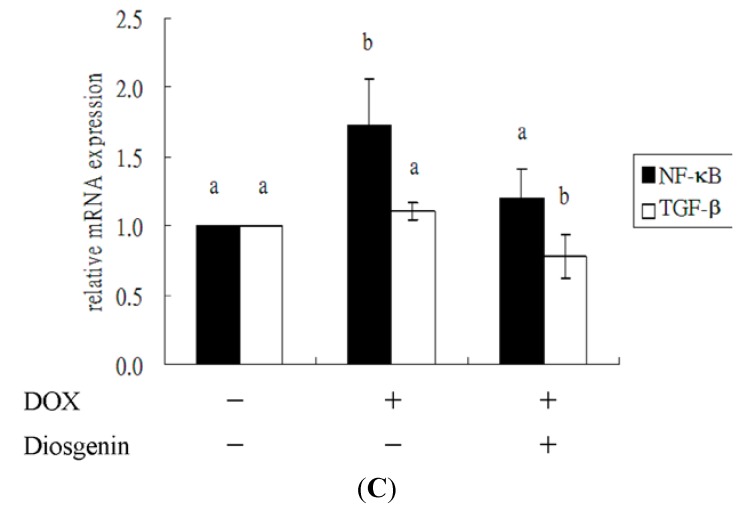
Effect of diosgenin on the activation of caspase-3 (**A**); the release of cytochrome c (**B**); and mRNA expressions of NF-κB and TGF-β (**C**) in heart tissues of mice treated with DOX for four weeks. Values are mean ± SD, *n* = 10. ^a,b^ Means in a row without a common letter differ, *p* < 0.05. The protein expressions of cytochrome c (cyto. c) from mitochondrial (Mito., line 1–2) and cytosolic (Cyto., line 3–4) fractions. COX IV and β-actin, respectively, served as an internal control of Mito. and Cyto. fractions. The protein levels above the figures represent relative density of the bands normalized to COX IV (*upper panel*) or β-actin (*bottom panel*). Determined expression of the protein was subsequently quantified by densitometric analysis with that of control being 1.00-fold, as shown just below the gel data. Results are representative of at least three independent experiments.

To investigate whether the protective effect of diosgenin against DOX occurred because it inhibited apoptotic pathways, we further studied the change in the expression of caspase-3, one marker of apoptosis, in the heart tissue (Figure 2A). Caspase-3 is a cytosolic protein that exists normally as an inactive precursor with a higher molecular weight (about 32 kDa). It is cleaved proteolytically into low molecular weights (11, 17, and 20 kDa) when a cell undergoes apoptosis [31]. In this study, treatment with DOX induced significantly an increase in cleavage of caspase-3 to 1.53-fold of that of control. In the diosgenin co-treated group, the active fragment was decreased (Figure 2A). As shown in Figure 2B (lane 2), DOX increased significantly the cytosolic concentration of cytochrome c, with a concomitant decrease in the mitochondria, implicating mitochondrial dysfunction due to DOX toxicity [32]. Quantitative data showed that the cytosolic concentration of cytochrome c was increased to 1.87-fold of that of control in the heart tissues of the DOX-treated mice. This effect was significantly suppressed in the DOX plus diosgenin group (lane 3, Figure 2B). The DOX-induced release of cytochrome c from mitochondria was inhibited by 81.6% in the DOX plus diosgenin group. In addition, mRNA expressions of NF-κB and transforming growth factor-beta (TGF-β), two factors that are related to inflammation, were measured by real-time PCR. DOX treatment augmented the cardiac mRNA expression of NF-κB, but not TGF-β. However, when mice were co-treated with diosgenin, concomitant decreases in their expressions were observed, as compared to the DOX treated group (*p* < 0.05).

### 3.5. Diosgenin Restored DOX-Regulated PKA and p38 Activation

**Figure 3:**
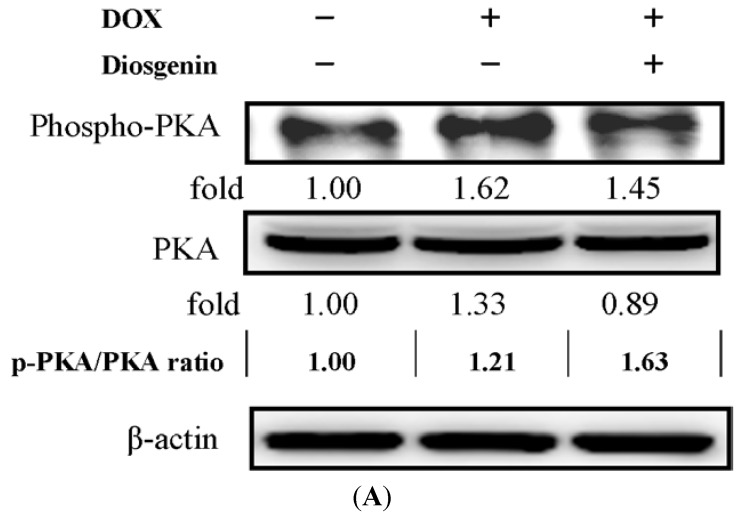

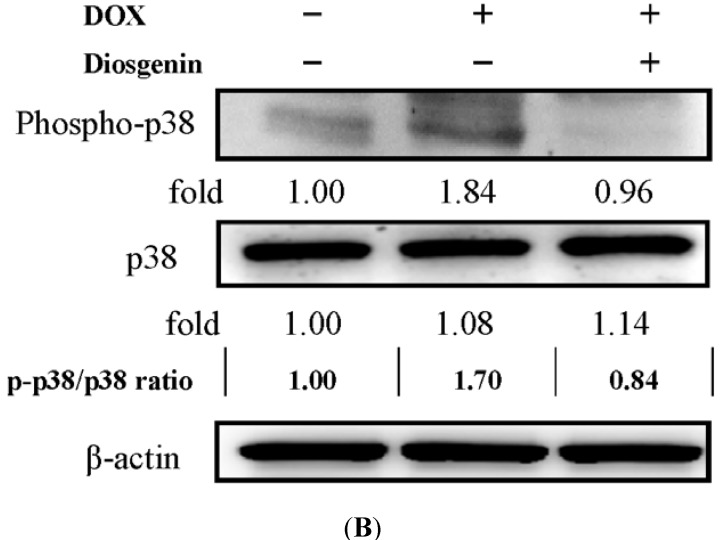
Effect of diosgenin on the activation of PKA (**A**) and p38 (**B**) in heart tissues of mice treated with DOX for four weeks. β-actin was served as an internal control of protein level. Determined expression of the protein was subsequently quantified by densitometric analysis with that of control being 1.00-fold, as shown just below the gel data. Results are representative of at least three independent experiments. The ratio between phosphorylated and total forms was indicated.

Previous studies found that cAMP-protein kinase A (PKA) pathway promoted cardiomyocyte survival [33]. It has also been shown that p38 mitogen-activated protein kinases (MAPK) pathway is involved in the DOX-induced cardiac oxidative, inflammatory and apoptotic reactions [34]. Consequently, we examined the phosphorylation of PKA and p38, and their total protein levels by Western blotting. As shown in Figure 3, the expressions of phospho-PKA, PKA (Figure 3A), phospho-p38, and p38 (Figure 3B) showed significant increases of about 1.62-, 1.33-, 1.84-, and 1.08-folds, respectively, in the DOX treated group, whereas diosgenin treatment modulated the activation of both protein kinases that were elevated in the presence of DOX (Figure 3).

## 4. Discussion

In cancer therapy, especially chemotherapy, DOX has made substantial help in cancer treatment. However, many side effects limit its benefits. Cardiotoxicity, a major side effect of DOX, can be observed in clinical patients and animal studies. The present study examined the possible protective effect of diosgenin, one bioactive compound in yam, on cardiac function in a mouse model of DOX-induced cardiomyopathy. Our data indicated that co-treatment of diosgenin with DOX for four weeks improved cardiac function during the DOX-induced cardiomyopathy, as demonstrated by improvements in body weight, heart weight, as well as in functional parameters including heart rate, blood pressure, and serum levels of LDH, CPK and CK-MB (Table 2). The preservation of heart function was associated with a decrease in the level of oxidative stress (Table 3) and apoptosis (Figure 2A,B) in cardiomyocytes as well as a significant decrease in inflammation status (Figure 2C). In animal models, a similar response has been reported [9,26].

DOX is well known for its cardiac toxicity during chemotherapy for cancer patients. The DOX-induced heart failure has been characterized by the generation of free radicals in the heart tissue [35,36]. In Table 3, the results showed that the activity of SOD was significantly decreased in the DOX-treated animals and the co-treatment of diosgenin reversed the SOD activity with a concomitant rise in the activity of GPx. GSH level was also lowered significantly in the DOX-treated animals, while diosgenin treatment showed a significant increase in GSH level (Table 3). The observed decrease in the activities of SOD and GPx in the DOX-treated animals supports the hypothesis that their decrease are possibly due to excessive use to overcome ROS production. These findings indicate the promising role of diosgenin as a cardioprotective agent against the DOX-induced cardiotoxicity. The antioxidant supplements have been suggested to patients with cancer to enhance the benefits of treatment. Antioxidants may also reduce certain types of toxicity associated with chemotherapy [37]. Diosgenin is a potential antioxidant molecule that has a helpful effect on the heart against DOX.

Currently, combinations of anticancer drugs with new agents are being investigated to explore a significant prognostic benefit and improve clinical response. To date, diosgenin has been reported to be very effective against arthritis, gastrointestinal disorders, cardiovascular dysfunction, inflammation and cancer [17,23,38,39,40]. The present study established the cardioprotective effects of diosgenin in the model of DOX-induced mice. The combinations of diosgenin with DOX indeed improved the DOX-induced cardiac toxicity. Further works are needed to clarify whether diosgenin might modulate the cancer killing effect of DOX *in vivo*. Diosgenin has been shown to exert anti-cancer effects against a wide variety of tumor cells, including colorectal cancer [41], leukemia [42], breast cancer [43], and liver cancer [44]. Moreover, diosgenin potentiated the apoptotic effects of DOX and paclitaxel in human hepatocellular carcinoma HUH-7 cells [44]. Sun *et al.*, indicated that dioscin, the glycoside form of diosgenin, is a potent multidrug resistance reversal agent in the multidrug resistant cell line human hepatocellular carcinoma (HepG2)/DOX, and may be a potential adjunctive agent for tumor chemotherapy [45]. This suggests diosgenin is effective in modulating the anti-cancer effect of DOX *in vivo*. Future experiments will be carried out to test this possibility and the detail mechanism.

A major adverse side effect associated with DOX use in the clinic is the occurrence of cardiomyopathy and heart failure. Therefore, several reports suggest that the DOX-induced apoptosis plays an important role in cardiotoxicity that is linked to the formation of ROS [46]. ROS production or oxidative stress promotes apoptosis, necrosis and autophagy in cardiomyocytes [47]. Caspase-3 activity can also be influenced by DOX [11]. Antioxidant enzymes form the first line of defense against cardiac tissue damage, and an increased oxidative stress may be due to depletion of antioxidants as reported earlier [36]. In the present study, the DOX-treated group showed an increase in active caspase-3 expression, indicating enhance effect of caspase-3 activity in apoptosis (Figure 2A). After treatment with diosgenin, we observed a significant decrease in the cleavage of caspase-3 (Figure 2A), which is consistent with previous investigations [7,9].

To understand the mechanisms of the diosgenin-mediated protection in the DOX-induced deterioration of cardiac function, we assessed the level of cGMP and cAMP in the heart tissue (Figure 1A). The results of our study showed that diosgenin restored the cGMP and cAMP levels (Figure 1A) as well as decreased the PDE5A expression (Figure 1B) in the heart. cGMP and cAMP are intracellular secondary messengers that mediate multiple cellular functions and morphological processes in the heart, including cardiac protection [48] against apoptosis and hypertrophy [49,50]. At physiological conditions, cGMP and cAMP are inactivated through hydrolysis degradation via PDE. The PDEs vary in their substrate specificity for cGMP and cAMP, among which PDE5 is specific for cGMP, and PDE3 has a mixed specificity for both cAMP and cGMP [51]. In a murine hypertension model, oral supplement of a PDE5A inhibitor, sildenafil, prevents and reverses cardiac hypertrophy, which is mediated by an activation of cGMP-dependent protein kinase [52]. These results suggest that treatment with PDE5 inhibitors might become a promising therapeutic intervention for preventing the DOX-induced cardiotoxicity, which is consistent with some previous studies [53,54]. cAMP promoted cardiomyocyte survival via an effect mediated through the cAMP pathway and the extracellular signal-regulated kinase activation [55]. Our results demonstrated that diosgenin can rescue the DOX-induced cardiac cell death effect leading to a down-regulation of cardiomyocyte contractility via cAMP-PKA pathway (Figure 1A and Figure 3A).

Many previous studies revealed that MAPKs play a crucial role in the development of hypertrophy processes such as inflammation and fibrosis, and p38 MAPK can act as a therapeutic target [34,56]. It has also been reported that DOX can promote cardiac oxidative, inflammatory and apoptotic reactions via activation of both NF-κB and MAPK pathways in the heart [57,58]. Our present study showed that diosgenin declined the mRNA level of NF-κB (Figure 2C) and down-regulated the expression of p-p38 (Figure 3B). Pharmacological inhibition of p38 MAPK protects cardiac myocytes from apoptosis during simulated I/R *in vitro*, indicating that p38 MAPK functions as a pro-apoptotic signaling effector [59]. It is therefore possible that the inhibitory effect of diosgenin on the DOX-induced cardiotoxicity was conducted via inactivating p38 that subsequently led to a reduction in the apoptotic signaling. However, their relevance needs to be demonstrated.

## 5. Conclusions

DOX-induced cardiomyopathy is an important public health concern because this may limit its therapeutic use for clinical patients. The search for cardioprotective agents will continue to rely on increasing our understanding of the mechanisms of the DOX-induced cardiotoxicity and how to counteract and overcome it. Our results suggest that diosgenin could be a promising agent to prevent the DOX-induced cardiomyopathy via its antioxidant, cGMP pathway, anti-apoptosis and anti-inflammation effects.

## Supplementary Materials

Supplemental Figure S1—Effect of diosgenin on TBARS (A) and GSH (B) levels in kidney tissues of mice treated with DOX for 4 weeks.
