# Supplementary material for: In Vivo Protective Effects of Diosgenin against Doxorubicin-Induced Cardiotoxicity
Source: Nutrients. 2015 Jun 17;7(6):4938–54. doi: 10.3390/nu7064938 (PMC4488824; doi:10.3390/nu7064938)
Supplement: Supplementary file 1 [file nutrients-07-04938-s001.docx]

**Supplementary Materials**

| 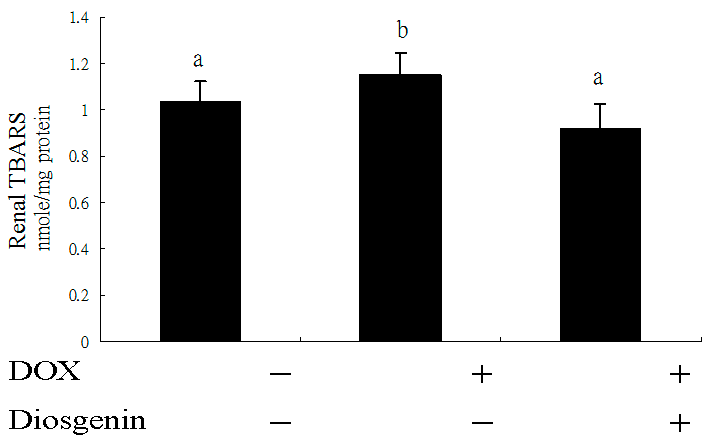 | 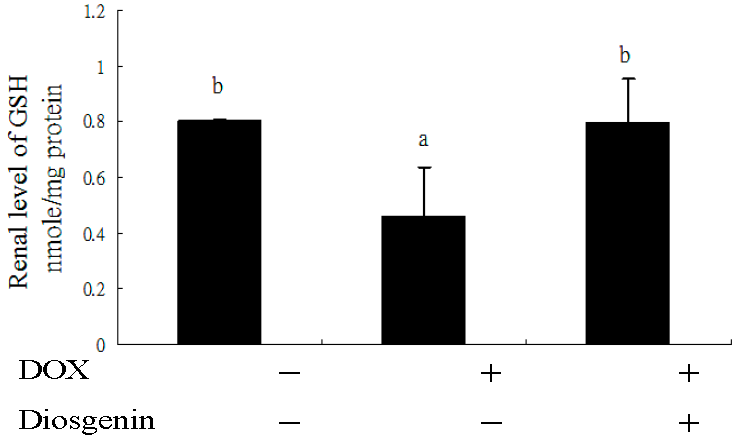 |
| --- | --- |
| (**A**) | (**B**) |

**Figure S1.** Effect of diosgenin on TBARS (**A**) and GSH (**B**) levels in kidney tissues of mice treated with DOX for 4 weeks. Values are mean ± SD, *n* = 10. ^a, b^ Means in a row without a common letter differ, *p* < 0.05.

© 2015 by the authors; licensee MDPI, Basel, Switzerland. This article is an open access article distributed under the terms and conditions of the Creative Commons Attribution license (http://creativecommons.org/licenses/by/4.0/).
